# A Remedy for Hæmoptysis

**Published:** 1872-12

**Authors:** 


### A REMEDY FOR HÆMOPTYSIS.

Dr. Holden states, in the Medical Record, that "He desires to call the attention of the profession to a method of treatment of hæmoptysis, which, while most simple and efficacious, he has not seen described by any one; namely, the throwing of the atomized vapor of a saturated solution of gallic acid directly into the mouth and throat. He has repeatedly found the most gratifying success to follow this treatment at once, even in cases of profuse hæmorrhage. Unlike other styptics thus administered, it quiets the spasmodic cough, which seems the direct result of the presence of the blood, requires but a moment to prepare, and aside from its efficacy, it inspires immediately the confidence of the patient. For about two years he has adopted this method, and has been surprised that no similar experience has found its way into the medical journals. His habit has been to have an atomizer and bottle of gallic acid always at hand, and when summoned hastily, to mix the acid in a tumbler of cold water, and use even without waiting for the excess of acid to subside. It has proved successful in several cases where blood was streaming from the mouth with every expiration."—*Medical and Surgical Reporter.*

